# Visible-Light Activated Titania and Its Application to Photoelectrocatalytic Hydrogen Peroxide Production

**DOI:** 10.3390/ma12244238

**Published:** 2019-12-17

**Authors:** Tatiana Santos Andrade, Ioannis Papagiannis, Vassilios Dracopoulos, Márcio César Pereira, Panagiotis Lianos

**Affiliations:** 1 Department of Chemical Engineering, University of Patras, 26500 Patras, Greece; tsandrade@live.com (T.S.A.); ion.papg@gmail.com (I.P.); 2 Institute of Science, Engineering, and Technology, Universidade Federal dos Vales do Jequitinhonha e Mucuri, Campus Mucuri, 39803–371 Teófilo Otoni, Minas Gerais, Brazil; mcpqui@gmail.com; 3 FORTH/ICE–HT, P.O. Box 1414, 26504 Patras, Greece; indy@iceht.forth.gr

**Keywords:** hydrogen peroxide, TiO_2_, CdS, CdSe, photoelectrocatalysis, photocatalytic fuel cells, photo fuel cells

## Abstract

Photoelectrochemical cells have been constructed with photoanodes based on mesoporous titania deposited on transparent electrodes and sensitized in the Visible by nanoparticulate CdS or CdS combined with CdSe. The cathode electrode was an air–breathing carbon cloth carrying nanoparticulate carbon. These cells functioned in the Photo Fuel Cell mode, i.e., without bias, simply by shining light on the photoanode. The cathode functionality was governed by a two-electron oxygen reduction, which led to formation of hydrogen peroxide. Thus, these devices were employed for photoelectrocatalytic hydrogen peroxide production. Two-compartment cells have been used, carrying different electrolytes in the photoanode and cathode compartments. Hydrogen peroxide production has been monitored by using various electrolytes in the cathode compartment. In the presence of NaHCO_3_, the Faradaic efficiency for hydrogen peroxide production exceeded 100% due to a catalytic effect induced by this electrolyte. Photocurrent has been generated by either a CdS/TiO_2_ or a CdSe/CdS/TiO_2_ combination, both functioning in the presence of sacrificial agents. Thus, in the first case ethanol was used as fuel, while in the second case a mixture of Na_2_S with Na_2_SO_3_ has been employed.

## 1. Introduction

Titanium dioxide (Titania, TiO_2_) is the most popular photocatalyst, and this is justified by the fact that it is stable, it can be easily synthesized and deposited on solid substrates as mesoporous film of several types of nanostructures, it is considered non–toxic and, most of all, it has very good electronic properties [1,2,3,4]. Thus, it is characterized by relatively high charge-carrier mobility, while its valence band has a high oxidative potential capable of carrying out most oxidative reactions. Its only disadvantage is that its light absorption is limited in the UV. For this reason and in order to expand its light absorption range, titania has been combined with visible-light-absorbing sensitizers [5,6,7,8,9,10,11,12,13,14,15,16,17]. Dyes as well as organometal halide perovskites have been very successful as sensitizers of titania, but their functionality is limited to specific organic or solid-state environments exclusively applied to solar cells [5,6]. In aqueous environments, only inorganic sensitizers have offered acceptable performance, and among those, only a few II–VI semiconductors have led to interesting scenarios [7,8,9,10,11,12,13,14,15,16,17]. Indeed, sensitization of mesoporous titania by nanoparticulate CdS and CdSe has been repeatedly studied, righteously offering efficient sensitization and application in a variety of photocatalytic and photoelectrocatalytic systems [7,8,9,10,11,12,13,14,15,16,17]. Furthermore, formation of nanoparticulate metal sulfides within the titania mesoporous structure is very easy and necessitates only “soft” chemistry techniques. In the present work, we have followed this established route for titania sensitization in order to construct photoelectrochemical devices capable of producing a valuable solar fuel, i.e., hydrogen peroxide.

The study of hydrogen peroxide has become very popular in recent years [18]. In addition to its well-known pharmaceutical applications, hydrogen peroxide is a key substance in advanced oxidation processes for water treatment, either alone or in combination with iron ions in Fenton processes [19,20,21]. Hydrogen peroxide is also a means of energy storage in the form of chemical energy since it can be used as fuel in hydrogen peroxide fuel cells or as oxidant for the operation of several fuel cell types [22,23,24,25,26]. Hydrogen peroxide can be easily handled since it is soluble in water and its consumption simply results to oxygen or water production. It is then obvious that hydrogen peroxide is indeed a valuable fuel, and for this reason it is worth producing it in a sustainable manner [27,28,29,30]. Photoelectrocatalysis, which can directly exploit solar energy and employ biomass-derived wastes as fuel may thus offer the means for sustainable hydrogen peroxide production. This route is investigated in the present work, and in this sense it constitutes a novelty in spite of the fact that the materials used to make electrodes are well known and frequently used in the past.

**Figure 1:**
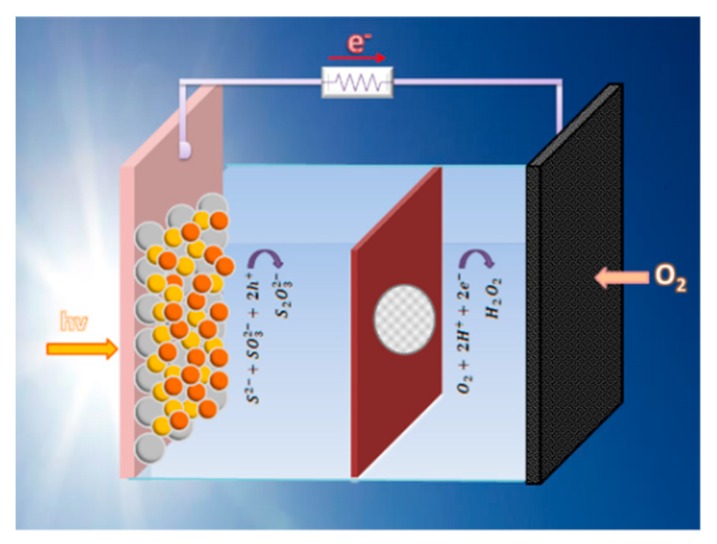
Schematic representation of the reactor used in the present work. The oxidation reaction in the anode compartment corresponds to the case of the S^2−^/SO_3_^2−^ electrolyte and the CdSe-enriched photoanode.

The purpose then of the present work is to study the production of hydrogen peroxide by means of photoelectrocatalysis. To this goal, we have used a photoelectrochemical cell employing a photoanode carrying visible–light–sensitized nanoparticulate titania. These cells work as self-running Photo(catalytic) Fuel Cells (PFC), operating without external bias by photocatalytically oxidizing a fuel, which may as well be a waste. This offers a double environmental benefit and ensures sustainability. Hydrogen peroxide has been produced by atmospheric oxygen reduction. The photoanode produced the current while an air–breathing cathode produced hydrogen peroxide. This is schematically represented in Figure 1, while details are discussed in the following sections.

## 2. Materials and Methods 

### 2.1. Materials

Unless otherwise specified, all reagents were obtained from Sigma–Aldrich and were used as received. Thus, Fluorine-doped Tin Oxide electrodes (FTO, 8 ohm/square) were purchased from Pilkington North America (Toledo, OH, USA), carbon cloth (CC) from Fuel Cell Earth (Wobum, MA, USA), carbon black (CB) from Cabot Corporation (Vulcan XC72, Billerica, MA, USA), and Nafion membrane from Ion Power, Inc (Newcastle, DE, USA).

### 2.2. Construction of the Photoanode

#### 2.2.1. Deposition of the Titania Film

The photoanode electrode was constructed by the following procedure. An FTO glass was cut in the appropriate dimensions and was carefully cleaned first with soap and then by sonication in acetone, ethanol, and water. A compact titania layer was first deposited on the clean electrode by a sol gel procedure. A precursor solution was prepared by mixing 3.5 g of Triton X-100 with 19 mL of ethanol, to which 3.4 mL of glacial acetic acid and 1.8 mL of titanium isopropoxide was added under stirring. This solution was used for dipping FTO electrodes, which were patterned by covering with tapes the back side and the front side parts, which should remain clear. Then, it was calcined up to 500 °C. This was repeated once, to ensure a complete coverage of the active electrode area. Next, a mesoporous titania layer was deposited on this compact layer by doctor blading, using a paste composed of Degusa P25 nanoparticles and prepared by a standard procedure based on [31]. The mesoporous film was calcined at 550 °C. This procedure was repeated once again to ensure that a mesoporous film of around 10 µm thick was obtained. Film thickness was approximately determined by SEM. The active area of the titania film was 1 cm^2^ (1 cm × 1 cm).

#### 2.2.2. Application of CdS on the Titania Film 

A fresh titania film was sensitized by CdS nanoparticles by the SILAR (Successive Ionic Layer Adsorption and Reaction) method [7,32,33] using 0.1 M cadmium nitrate as Cd^2+^ and 0.1 M sodium sulfide as S^2−^ source. Ten SILAR cycles were sufficient to cover titania with the yellow CdS layer. This method does not produce a separate CdS layer, but rather, CdS nanoparticles are formed within the titania mesoporous structure [7]. At the end, the film was first dried in a nitrogen stream and then for a few minutes in an oven at 70 °C. This electrode was either used as CdS/TiO_2_/FTO photoanode or as substrate for the next step of CdSe deposition. 

#### 2.2.3. Addition of the CdSe Layer and the ZnS Protective Layer

CdSe was added on the top of CdS by a chemical bath deposition (CBD), as in previous publications [8,32]. CdSe is formed in a period of about 4–5 h at low temperature (in a refrigerator). The CdS-sensitized TiO_2_ film was immerged face-up in a precursor solution containing 27 mM of sodium selenosulphate (Na_2_SeSO_3_) as a source of Se^2−^, and 27 mM of cadmium sulfate as a source of Cd^2+^. The precursor solution was prepared by observing the following protocol. An aqueous solution of 80 mM Se powder was first prepared in the presence of 0.2 M Na_2_SO_3_ by continuous stirring and refluxing at 80 °C. The procedure lasted about 15 h and was carried out overnight. The obtained solution, denoted in the following as sol A, actually aimed at the formation of the above sodium selenosulphate (Na_2_SeSO_3_). Then, an aqueous solution of 0.12 M nitrilotriacetic acid trisodium salt was prepared, denoted as sol B. Finally, an aqueous 80 mM CdSO_4_·8/3H_2_O solution was also prepared, denoted as sol C. Sol B was mixed with an equal volume of sol C, and the obtained mixture was stirred for a few minutes. The combination of sol B with sol C leads to the formation of a complex, which is used as precursor for slow release of cadmium ions. Finally, two parts of this last mixture were mixed with one part of sol A, and the thus obtained final mixture was used for CBD. At the end of 4–5 h, the CdS/TiO_2_ film was red-colored. The thus obtained film was again first dried in a N_2_ stream and then in an oven at 70 °C. It is a common practice to stabilize the CdSe film by a top layer of ZnS [32], which was actually done in the present work. Thus a ZnS layer was finally deposited on the top by 2 SILAR cycles using zinc nitrate as Zn^2+^ source and N_2_S as S^2−^ source, followed by drying as above. This procedure yielded ZnS/CdSe/CdS/TiO_2_/FTO photoanode electrodes with a broad range of light absorption.

### 2.3. Construction of the Counter Electrode

The cathode electrode was a carbon cloth covered on one side with two layers of carbon black (CB/CC). The deposition of the CB layer was made by using a paste made by the following recipe: 300 mg of CB was mixed with 8 mL of twice-distilled water under vigorous stirring with a mixer (more than 4000 rpm), until a viscous paste was obtained. To this mixture 0.1 mL of PTFE (60% in water) was added as a hydrophobic binder and was again vigorously stirred. This paste was applied on the carbon cloth with a spatula, dried in an oven at 80 °C, and then sintered at 340 °C. The procedure was repeated once to reach a quantity of CB equal to 1 mg per cm^2^. The active area of the electrode was 1 cm^2^, as in the case of the photoanode.

### 2.4. Description of the Reactor

The reactor was a home-made device based on Plexiglas, which was divided into two compartments by a Nafion membrane, as schematically shown in Figure 1. The capacity of each compartment was 5 mL. It had two windows which were sealed by the two electrodes (photoanode and cathode). It was filled with various electrolytes appropriate for each particular case, as described below. The active area of each window was 1 cm^2^, fitting the above electrodes. Illumination of the photoanode was made with a Xe lamp, which provided approximately 100 mW cm^−2^ at the position of the catalyst. Light entered through the transparent FTO electrode.

### 2.5. Measurements

The quantity of produced H_2_O_2_ was spectroscopically determined by its concentration in the aqueous electrolyte of the cathode compartment. This was done by a standard method analytically described in the Supplementary File. Photoelectrochemical measurements were made with the support of an Autolab potentiostat PGSTAT128N (Metrohm Autolab B.V., Utrecht, The Netherlands). Reflection–Absorption spectra were recorded with a Shimadzu UV–2600 absorption spectrophotometer equipped with an integration sphere. Field-Emission Scanning Electron Microscopy images (FESEM) were obtained with a Zeiss SUPRA 35 VP device.

## 3. Results and Discussion

### 3.1. Characterization of Electrodes

**Figure 2:**
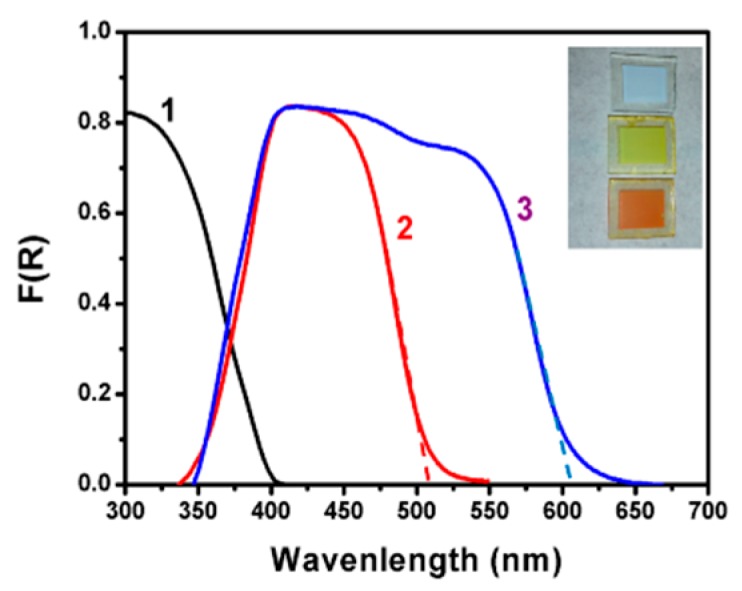
Reflection absorption spectra of various photoanode electrodes: (1) TiO_2_/FTO; (2) CdS/TiO_2_/FTO; and (3) CdSe/CdS/TiO_2_/FTO. In curves (2) and (3), the background including titania absorption has been subtracted for better presentation of the spectra. The tangential dashed lines give the average value of the band gap, i.e., 510 nm (2.43 eV) for the CdS/TiO_2_/FTO film and 610 nm (2.03 eV) for the CdSe/CdS/TiO_2_/FTO film. Insert: Film photographs.

As already discussed, the photoelectrochemical setup used in the present work employed three photoanode electrodes based on mesoporous TiO_2_ deposited on FTO transparent electrodes: Titania alone (TiO_2_/FTO), CdS-sensitized titania (CdS/TiO_2_/FTO), and a film with additional CdSe sensitizer on the top (CdSe/CdS/TiO_2_/FTO). The light absorption range of each film is given by the reflection–absorption spectra of Figure 2. The CdS/TiO_2_/FTO film approximately absorbed photons up to about 510 nm, corresponding to a band gap of 2.43 eV. The maximum photocurrent density expected for a photoanode carrying such a film and for solar radiation equal to 1 sun (100 mW cm^−2^) can be calculated from published charts [2,34] and is around 7 mA cm^−2^. Correspondingly, the CdSe/CdS/TiO_2_/FTO film absorbed photons up to 610 nm with a band gap of 2.03 eV and maximum current density approximately equal to 13 mA cm^−2^. Photoanodes made with such combined semiconductors may then yield substantial current densities appropriate for practical applications.

**Figure 3:**
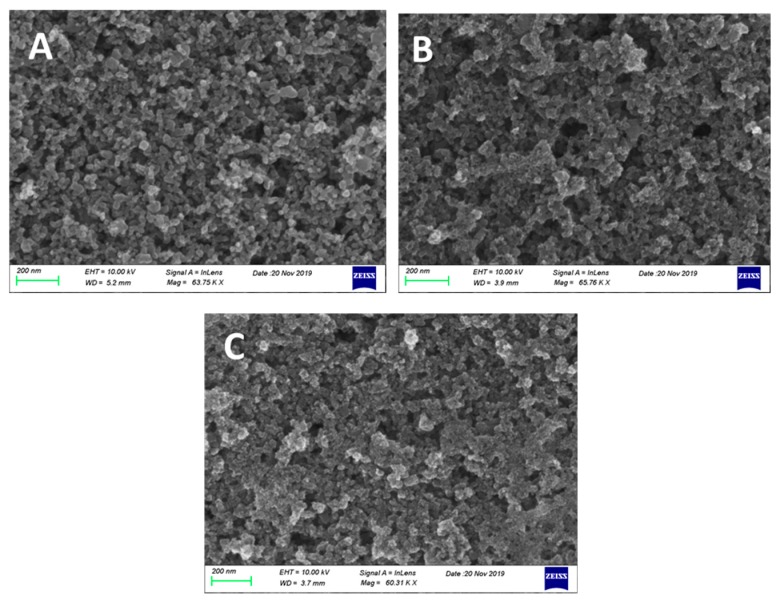
FESEM images of the three photoanode films: (**A**) TiO_2_/FTO; (**B**) CdS/TiO_2_/FTO; and (**C**) CdSe/ CdS/TiO_2_/FTO. The scale bar is 200 nm in all cases.

CdS and CdSe nanoparticles were synthesized, as explained in the Experimental section, by reaction of Cd^2+^ cations with S^2−^ anions within the pores of the mesoporous titania film. For this reason, they do not form separate layers, but they are detected inside the titania mesostructure. This is known from previous works [7,8,15,17,35,36], but can be also verified by the following FESEM images shown in Figure 3. Figure 3A shows a characteristic image of nanoparticulate Titania P25. TiO_2_ nanoparticles range in sizes around 20–30 nm. Careful observation and comparison of the three images shows the formation of new species within the mesoporous titania structure in going from pure titania to CdS/TiO_2_ (Figure 3B) and then to CdSe/CdS/TiO_2_ (Figure 3C). CdS and CdSe particles grow in much smaller sizes ranging below 10 nm. This intercalated formation of the chalcogenide semiconductors ensures close proximity between nanoparticles and subsequently efficient sensitization and charge carrier mobility. Some protective role of titania on chalcogenide nanoparticles cannot be excluded either.

**Figure 4:**
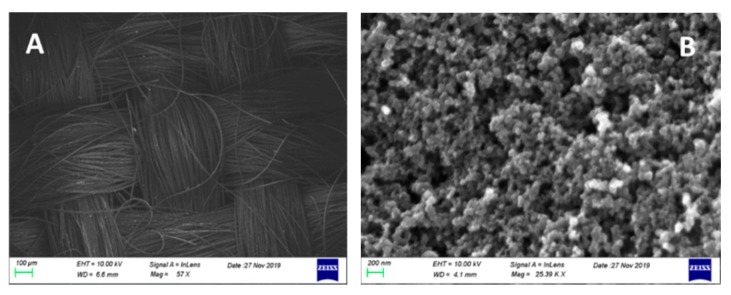
FESEM images of the carbon cloth (**A**) and the carbon black film (**B**). The scale bar is 100 μm and 200 nm, respectively.

The structure of the cathode electrodes (CB/CC) can be seen in the FESEM images of Figure 4, showing the weaving of carbon filaments making the carbon cloth and the mesostructure of the deposited carbon black. Carbon black covered all pores making an air-breathing (gas diffusion) electrode which sealed the electrolyte from any leak. This construction lasted for many hours and for many rounds of operation. 

**Figure 5:**
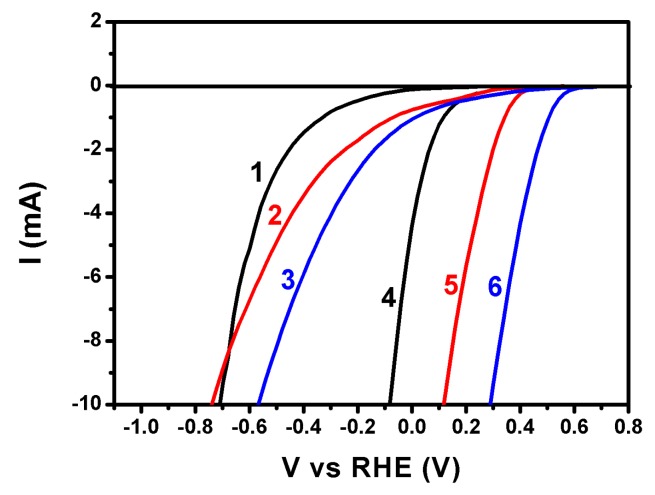
Polarization curves for an electrode made of carbon cloth alone (1,2,3) or carbon cloth carrying carbon black (4,5,6) in various 0.5 M aqueous electrolytes: (1,4) H_2_SO_4_; (2,5) Na_2_SO_4_; and (3,6) NaHCO_3_. A Pt foil was used as counter and an Ag/AgCl as reference electrode.

The capacity of the CB/CC electrode to carry out reduction reactions may be qualitatively appreciated by the polarization curves of Figure 5. These curves were obtained in a 3-electrode configuration using CB/CC or plain CC as working, a Pt foil as counter, and Ag/AgCl as reference electrode. Curves were traced in aqueous electrolytes containing 0.5 M of either H_2_SO_4_, Na_2_SO_4_, or NaHCO_3_. All curves have been plot vs. Reversible Hydrogen Electrode (RHE) by taking into account the pH value of each electrolyte, i.e., 1.0, 6.5, and 8.5, respectively, and by adding 0.2 V for the potential of the reference electrode. The importance of the presence of carbon black is first obvious by the fact that reduction reactions are obtained at negative potentials in its absence. In the presence of carbon black, the most favorable reduction was obtained in a carbonate electrolyte, where the reduction potential was the most positive of the three electrolytes. More positive reduction potential means higher intrinsic bias for electron flow from the photoanode to the cathode electrode, therefore, more efficient electrochemical process. Even though the data of Figure 5 are approximate and do not allow accurate quantitative results, they are sufficient to highlight NaHCO_3_ as the most promising of the three electrolytes presently tested. As it will be seen below, hydrogen peroxide production was indeed the highest in this electrolyte.

### 3.2. Current–Voltage Characteristics of Various Photo Fuel Cells

**Figure 6:**
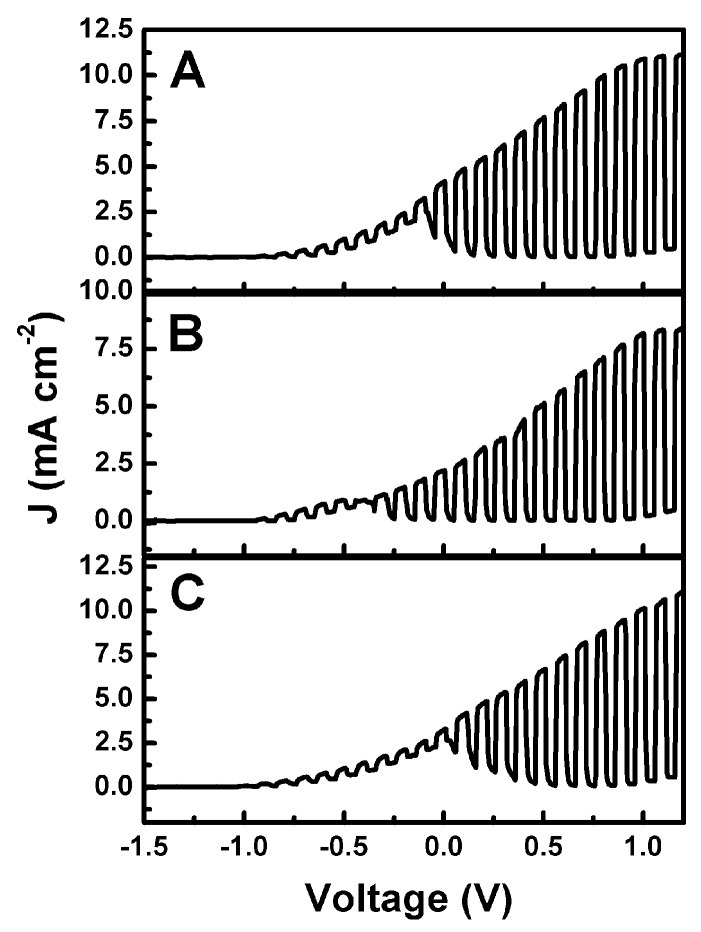
Photo Fuel Cell current density–voltage curves plot in the light chopping mode corresponding to the same electrolyte in the anode compartment (0.5 M aqueous NaOH + 5% w/w ethanol) and three different 0.5 M aqueous electrolytes in the cathode compartment: (**A**) H_2_SO_4_; (**B**) Na_2_SO_4_; and (**C**) NaHCO_3_.

The above described photoanode and cathode electrodes were used to operate various versions of a photo fuel cell. Figure 6 shows current–voltage characteristics of a two-compartment cell functioning with a CdS-sensitized photoanode (CdS/TiO_2_/FTO) and a CB/CC cathode. A Nafion membrane separated the two compartments. The anode aqueous electrolyte was 0.5 M NaOH with added 5% w/w ethanol. The cathode compartment contained either 0.5 M aqueous H_2_SO_4_, Na_2_SO_4_, or NaHCO_3_. Curves were plot in a two-electrode configuration in the light-chopping mode to reveal the conditions of photocurrent production. Maximum photocurrent density was obtained under forward bias and exceeded 10 mA cm^−2^ in all three cases. These values were higher than expected for the present CdS-sensitized TiO_2_ photoanode (i.e., larger than 7 mA cm^−2^, see above). This is due to the presence of ethanol and the ensuing current doubling phenomena [37]. Current doubling phenomena are always observed with photoelectrochemical cells functioning in the presence of an organic fuel, and they are reported in almost all of our related works. At zero bias, which is of interest in the present case, substantial photocurrent was produced in all three cases, therefore, production of hydrogen peroxide in a Photo Fuel Cell mode was monitored in all three cases, as will be detailed below. In the curves of Figure 6, there is interference to the photocurrent by a capacitance current due to adsorption of cations in the photoanode mesostructure [38]. This capacitance current appears only when plotting current–voltage curves and is of no importance for the rest of the measurements.

**Figure 7:**
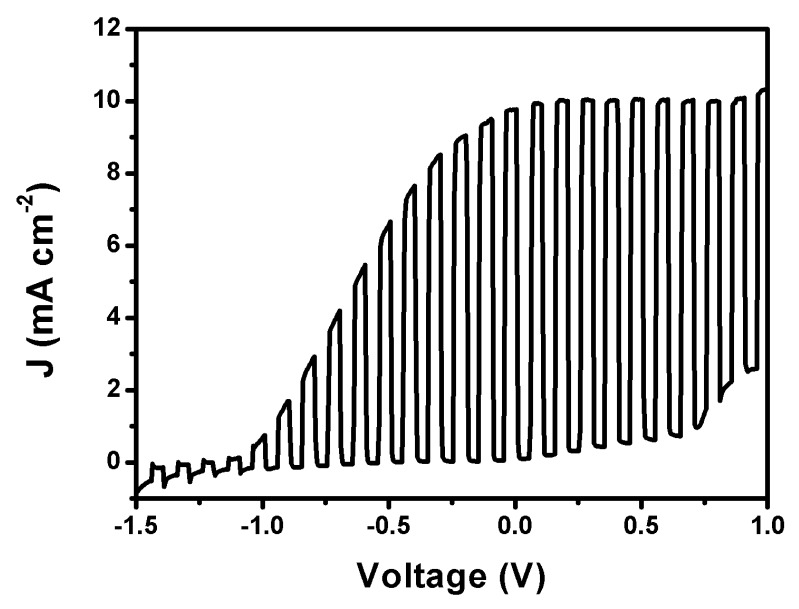
Photo Fuel Cell current density–voltage curve plot in the light chopping mode corresponding to 0.25 M Na_2_S + 0.125 M Na_2_SO_3_ aqueous electrolyte in the anode compartment and 0.5 M aqueous NaHCO_3_ electrolyte in the cathode compartment.

In another version of the Photo Fuel Cell, the photoanode carried the ternary semiconductor film, i.e., CdSe/CdS/TiO_2_/FTO. In fact, as explained in Section 2.2.3, there was an additional protective layer of ZnS deposited on the top by 2 SILAR cycles. This layer is not sufficient to change the spectroscopic characteristics of the photoanode. ZnS absorbs in the UV, and its role is only to protect the underlying film. Its addition is a common practice for such films [32]. The ternary photoanode is not stable in the NaOH+ethanol electrolyte. For this reason, it was employed in the presence of an aqueous mixture of Na_2_S with Na_2_SO_3_, which is frequently used with chalcogenide semiconductors and is considered a model for sulfur-containing water wastes. Figure 7 shows a current density–voltage curve obtained with a two-compartment Photo Fuel Cell comprising the above ternary semiconductor photoanode and a CB/CC cathode electrode. The two compartments were separated again by a Nafion membrane. The anode electrolyte was an aqueous 0.25 M Na_2_S and 0.125 M Na_2_SO_3_ mixture. The cathode electrolyte was an aqueous 0.5 M NaHCO_3_ solution. The choice of this last electrolyte was made, as it will be seen below, by the fact that it offers the highest hydrogen peroxide yield. The maximum photocurrent was within the expected range (i.e., no more than 13 mA cm^−2^, see above). Of course, in the present case, no current doubling phenomena were observed since there was no organic additive in the anode electrolyte. It is noteworthy that the short-circuit current density in the present case was larger than 10 mA cm^−2^, much larger than in any of the three cases of Figure 6. Here then there is an interesting case, very promising for practical applications. Both devices, i.e., the one carrying the CdS/TiO_2_/FTO photoanode and the one carrying the CdSe/CdS/TiO_2_/FTO photoanode, are useful for sustainable production of hydrogen peroxide. In the first case, a biomass-derived fuel may be used, while in the second case, a water waste containing sulfur products, for example from oil refineries, may be used.

### 3.3. Photoelectrocatalytic Hydrogen Peroxide Production

**Figure 8:**
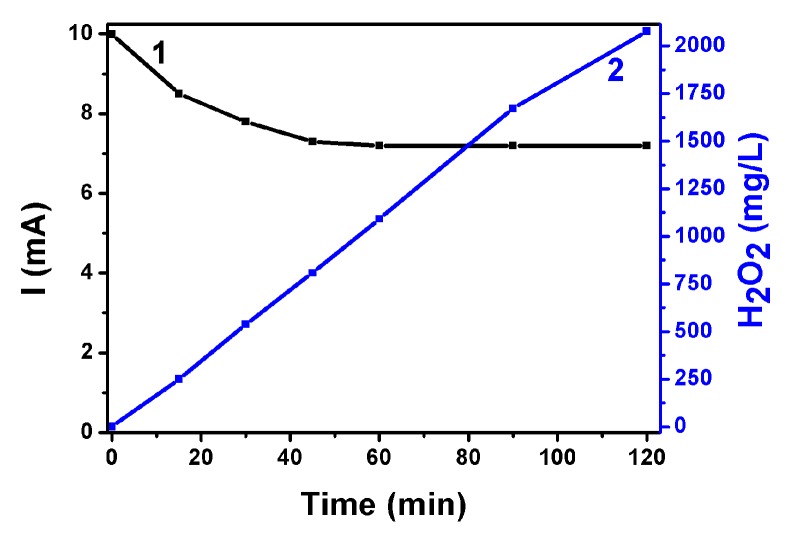
Evolution of the short-circuit current (1) and of the quantity of photoelectrochemically produced H_2_O_2_ (2) using a PFC operated with a ternary semiconductor photoanode. Anode electrolyte: Aqueous 0.25 M Na_2_S + 0.125 M Na_2_SO_3_. Cathode electrolyte: Aqueous 0.5 M NaHCO_3_.

Following the above characterization of electrodes and devices, hydrogen peroxide production has been monitored by PFC operation. The first system studied was the one operating with the ternary semiconductor photoanode (i.e., CdSe/CdS/TiO_2_/FTO), which produced the highest short-circuit photocurrent. The anode compartment contained an aqueous 0.25 M Na_2_S + 0.125 M Na_2_SO_3_ electrolyte, while the cathode electrolyte was an aqueous 0.5 M NaHCO_3_ solution. Production of H_2_O_2_ was monitored under potentiostatic–amperometric conditions at V = 0.0 V. The evolution of the short-circuit photocurrent is shown in Figure 8. The current density started at 10 mA cm^−2^. In the course of the experiment, it dropped to 7.2 mA cm^−2^, where it was finally stabilized. Hydrogen peroxide continuously evolved in a cumulative manner within a period of 120 min, as seen in Figure 8. An analysis of the H_2_O_2_ as a function of time and in relation with the current flowing through the cell is presented in Table 1. The second column of this table gives the evolution of the concentration of hydrogen peroxide in the carbonate electrolyte, while the fifth column gives the average molar rate of H_2_O_2_ production in the period of the corresponding time (first column). To a rough approximation, the rate did not much vary in the course of the present experiment. The molar rate can be associated with the equivalent current that should flow through the cell to produce hydrogen peroxide by reduction reactions. Hydrogen peroxide production may be described by the following scheme:

O_2_ + 2H^+^ + 2e^−^ → H_2_O_2_ (1)

It then takes 2 electrons to form one H_2_O_2_ molecule. Consequently, 1 µmole min^−1^ of a substance which is formed by 2 electrons per molecule, corresponds to 10^−6^ mole × 6.023 × 10^23^ molecules mole^−1^ × 2 × 1.602 × 10^−19^ C molecule^−1^ × (60 sec)^−1^ = 3.21 mA. The corresponding equivalent current is then calculated by multiplying the molar rate by 3.21, and the obtained values are listed in column 6. By dividing this current by the corresponding actual average current flowing through the cell over each time period, we obtained the corresponding Faradaic efficiencies for hydrogen peroxide photoelectrocatalytic production. The Faradaic efficiency was very high and reached 100%. This means that all current generated by the present Photo Fuel Cell was consumed to produce hydrogen peroxide without losses. The carbonate environment is responsible for such a high efficiency both in view of the data of Figure 5 and the related discussion, and because NaHCO_3_ is known to catalyze hydrogen peroxide formation [39,40]. When sulfate was used in the presence of carbonate, Faradaic efficiency was substantially lower. Indeed, Table 2 presents an equivalent analysis with that of Table 1 on data obtained by the same system as above but by substituting the NaHCO_3_ electrolyte by aqueous 0.5 M Na_2_SO_4_. The current flowing through the cell was much lower, and the Faradaic efficiency for hydrogen peroxide production did not pass 65%. Apparently, NaHCO_3_ electrolyte is much more interesting for Photo Fuel Cell operation with the ternary semiconductor photoanode in the presence of sulfide/sulfite electrolyte.

Hydrogen peroxide was also produced by the PFC, which used the binary semiconductor photoanode, i.e., CdS/TiO_2_/FTO, and ethanol as a fuel. In that case, data have been obtained for three different electrolytes in the cathode compartment and the results are presented in Table 3, Table 4 and Table 5. The first case involved the carbonate electrolyte, which led to the highest H_2_O_2_ production. The results are shown in Table 3. The current flowing through the cell was now lower, as expected in accordance to the data of Figure 3. As seen in the 7th column, the original current was 3.8 mA cm^−2^ and settled at 3.2 mA cm^−2^. The H_2_O_2_ concentration continuously increased and so did the molar rate. It is interesting that the Faradaic efficiency was very high and grew beyond 100%, verifying the catalytic effect that the carbonate electrolyte has on the electrocatalytic production of hydrogen peroxide [39,40]. In these works, this effect was studied mainly for cases of hydrogen peroxide production by water oxidation and is related with the intermediate formation of HCO_3_^−^ and its catalytic effect on water oxidation. Apparently, equivalent processes may take place during oxygen and water reduction. This phenomenon necessitates further study. It must be noted at this point that no hydrogen peroxide was detected in the anode compartment, suggesting that for the present cells hydrogen peroxide is an exclusive product of reduction processes. Likewise, it is also a product of photoelectrochemical processes since no hydrogen peroxide was detected in the dark. In the presence of Na_2_SO_4_ instead of NaHCO_3_, according to the data of Table 4, the catalytic effect was not present anymore and the Faradaic efficiency was substantially lower. Finally, when sulfuric acid was used as cathode electrolyte, the system showed a very poor behavior, as seen in Table 5, so this electrolyte was excluded from any further measurements. The higher short-circuit current recorded in the case of H_2_SO_4_ and Na_2_SO_4_ (7th column of Table 4 and Table 5) is in accordance with the data of Figure 6. The PFC in that case had an alkaline electrolyte (i.e., NaOH) in the anode compartment with a pH value around 13, while the pH in the cathode compartment was 1, 6.5, and 8.5 in the case of H_2_SO_4_, Na_2_SO_4_, and NaHCO_3_, respectively. In the case then of the first two electrolytes, a strong forward bias of chemical nature developed between the two electrodes, which justifies the higher currents at V = 0.

In conclusion, the above data show that a photoelectrochemical cell operating as a Photo Fuel Cell, i.e., without any external bias, can produce substantial quantities of hydrogen peroxide with high Faradaic efficiency reaching values higher than 100% in the presence of a carbonate electrolyte. Hydrogen peroxide was produced at the cathode electrode, which was a Pt-free inexpensive combination of a carbon cloth with carbon black. Both anode and cathode electrodes were characterized after use. Some of the deposited material leached off the electrodes, but their nanostructure remained the same as imaged in Figure 3 and Figure 4. This materials leach is mainly responsible for the drop of current during cell operation.

## 4. Conclusions

This work has shown that a Photo Fuel Cell can be constructed with a visible light responsive photoanode based on chalcogenide-semiconductors-sensitized mesoporous titania and a simple Pt-free cathode made of carbon cloth with deposited nanoparticulate carbon (carbon black). This cell functioned without any bias producing substantial current. The cathode functionality is based on atmospheric oxygen reduction, which leads to hydrogen peroxide production. This functionality was exploited in order to photoelectrochemically produce hydrogen peroxide. High Faradaic efficiencies have been reached for the production of hydrogen peroxide, which in the presence of NaHCO_3_ rose beyond 100% due to the catalytic effect of the latter. 

## Figures and Tables

**Table 1:** Analysis of hydrogen peroxide photoelectrocatalytic production rate in 0.5 M NaHCO_3_. Case of the ternary semiconductor photoanode.

Time	H_2_O_2_ Conc.	H_2_O_2_ Mass	Corresp. Molarity	Molar Rate	Equivalent Current	Current at Time	Average Current	Faradaic Efficiency
(min)	(mg/L)	(mg)	(μmol)	(μmol/min)	(mA)	(mA)	(mA)	%
0	0	0	0	––	––	10	–	––
15	250	1.25	36.8	2.45	7.87	8.5	9.3	91
30	538	2.69	79.1	2.64	8.46	7.8	8.9	95
45	808	4.04	119	2.64	8.48	7.3	8.7	98
60	1092	5.46	161	2.68	8.59	7.2	8.6	100
90	1672	8.36	246	2.73	8.77	7.2	8.6	102
120	2077	10.4	305	2.54	8.17	7.2	8.6	95

**Table 2:** Analysis of hydrogen peroxide photoelectrocatalytic production rate in 0.5 M Na_2_SO_4_. Case of the ternary semiconductor photoanode.

Time	H_2_O_2_ Conc.	H_2_O_2_ Mass	Corresp. Molarity	Molar Rate	Equivalent Current	Current at time	Average Current	Faradaic Efficiency
(min)	(mg/L)	(mg)	(μmol)	(μmol/min)	(mA)	(mA)	(mA)	%
0	0	0	0	–	–	6.5	–	–
30	201	1.01	29.6	0.98	3.16	6.1	6.3	50
47	329	1.65	48.4	1.03	3.30	5.1	5.8	57
77	594	2.97	87.4	1.13	3.64	4.7	5.6	65

**Table 3:** Analysis of hydrogen peroxide photoelectrocatalytic production rate in 0.5 M NaHCO_3_. Case of the binary semiconductor photoanode.

Time	H_2_O_2_ Conc.	H_2_O_2_ Mass	Corresp. Molarity	Molar Rate	Equivalent Current	Current at Time	Average Current	Faradaic Efficiency
(min)	(mg/L)	(mg)	(μmol)	(μmol/min)	(mA)	(mA)	(mA)	%
0	0	0	0	–	–	3.8	–	–
15	113	0.57	16.7	1.11	3.56	3.4	3.6	99
30	247	1.23	36.3	1.21	3.89	3.2	3.5	111
47	415	2.07	61.0	1.30	4.17	3.2	3.5	119
60	556	2.78	81.8	1.36	4.38	3.2	3.5	125
75	728	3.64	107	1.43	4.59	3.2	3.5	131
95	944	4.72	139	1.46	4.69	3.2	3.5	134

**Table 4:** Analysis of hydrogen peroxide photoelectrocatalytic production rate in 0.5 M Na_2_SO_4_. Case of the binary semiconductor photoanode.

Time	H_2_O_2_ Conc.	H_2_O_2_ Mass	Corresp. Molarity	Molar Rate	Equivalent Current	Current at Time	Average Current	Faradaic Efficiency
(min)	(mg/L)	(mg)	(μmol)	(μmol/min)	(mA)	(mA)	(mA)	%
0	0	0	0	–	–	5.1	–	–
18	115	0.58	16.9	0.94	3.00	5.1	5.1	60
40	279	1.40	41.0	1.03	3.28	5.2	5.1	64
47	352	1.76	51.8	1.10	3.52	5.2	5.2	68
85	705	3.53	104	1.22	3.90	4.9	5.0	78

**Table 5:** Analysis of hydrogen peroxide photoelectrocatalytic production rate in 0.5 M H_2_SO_4_. Case of the binary semiconductor photoanode.

Time	H_2_O_2_ Conc.	H_2_O_2_ Mass	Corresp. Molarity	Molar Rate	Equivalent Current	Current at Time	Average Current	Faradaic Efficiency
(min)	(mg/L)	(mg)	(μmol)	(μmol/min)	(mA)	(mA)	(mA)	%
0	0	0	0	–	–	5.1	–	–
30	20	0.10	2.9	0.10	0.31	6.2	6.3	5
83	70	0.35	10.3	0.12	0.39	5.6	5.9	7
125	125	0.63	18.4	0.15	0.47	4.9	5.3	9
142	152	0.76	22.3	0.16	0.50	4.6	4.7	11

## Supplementary Materials

The following are available online at https://www.mdpi.com/1996-1944/12/24/4238/s1, Detailed description of the determination of hydrogen peroxide in solution.
