# Supplementary material for: Visible-Light Activated Titania and Its Application to Photoelectrocatalytic Hydrogen Peroxide Production
Source: Materials (Basel). 2019 Dec 17;12(24):4238. doi: 10.3390/ma12244238 (PMC6947256; doi:10.3390/ma12244238)
Supplement: Supplementary file 1 [file materials-12-04238-s001.pdf]

materials

Supporting Information

Visible-Light Activated Titania and its Application to Photoelectrocatalytic Hydrogen Peroxide Production

Tatiana Santos Andrade, Ioannis Papagiannis, Vassilios Dracopoulos, Márcio César Pereira and Panagiotis Lianos

Determination of aqueous hydrogen peroxide

The quantity of the photoelectrocatalytically produced hydrogen peroxide can be monitored by the following procedure. First, it is necessary to make a standard solution of about 200 mg/L of aqueous H_2O_2. This concentration fits the spectroscopy data as it will be explained below. For this purpose, we used commercial 30% aqueous H_2O_2. This product was diluted 2000 times and the quantity of H_2O_2 in the diluted sample was determined by potassium permanganate titration. More specifically, the commercial product was first diluted 100 times. Then in a conic flask containing water acidified with H_2SO_4 (about 5 ml of sulfuric acid added to 150 ml of water), we added 7.5 ml of the diluted sample. This approximately makes a 2000 times dilution of the original sample. Then a solution of 20 mM potassium permanganate was added dropwise under stirring until a persistent pink color was obtained. The concentration of hydrogen peroxide in the conic flask was determined by the following formula

$$C_{H_2O_2} = 2.5C_{per}\frac{v}{V+v}$$

where C_{per} is the concentration of the added permanganate solution (20 mM in the present case), v its volume and V is the volume of the solution before permanganate addition. The multiplication factor 2.5 comes from the well-known reaction scheme

$$2MnO_4^- + 5H_2O_2 + 6H^+ \rightarrow 2Mn^{2+} + 5O_2 + 8H_2O$$

which relates 2 permanganate molecules with 5 hydrogen peroxide molecules. The above titration allowed us to determine the exact concentration of hydrogen peroxide in the diluted solution and thus allowed us to make a standard aqueous H_2O_2 solution of 200 mg/L. In other words, permanganate titration was used in order to determine H_2O_2 concentration in the original commercial sample and thus safely prepare a 200 mg/L standard aqueous H_2O_2 solution.

The next step was to make a calibration curve with the help of potassium titanium oxalate (PTO). For this purpose and by following literature suggestions, a second standard aqueous solution was prepared, containing 25 mM PTO and 1M H_2SO_4. In 1 cm cuvette, we mixed 1 ml of the standard PTO solution with 0.2, 0.4, 0.6, 0.8 or 1.0 ml of the standard H_2O_2 solution. The mixture was completed by adding water so as the total volume of the water mixture to be always 2 ml. The mixture became yellow after mixing the components and gave a UV-Vis absorbance, which peaked at 400 nm. The absorbance was related with the nominal H_2O_2 concentration and produced the diagram of **Figure S1**. It is seen that with a solution of 200 mg/L H_2O_2, the corresponding absorbance remained in the range of accurate measurement by a UV-Vis spectrophotometer and this is the reason that this concentration was chosen as a standard. For the same reason, 25 mM of PTO was also chosen as a standard.

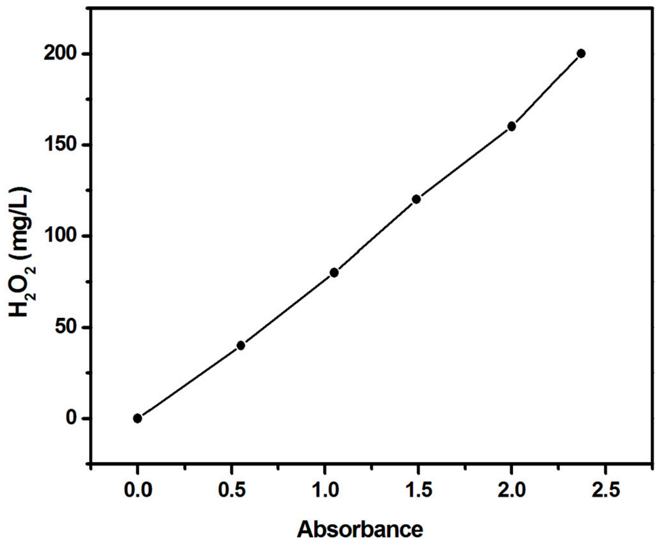

Figure S1. Nominal H_2O_2 concentration as a function of light absorbance at 400 nm. Each sample was made by mixing the same PTO solution with H_2O_2 solutions of various concentrations.

Once the calibration curve is made, we are ready to determine H_2O_2 concentration in any given sample. 1 ml of the sample is mixed with 1 ml of PTO standard solution and the corresponding hydrogen peroxide concentration is obtained by relating the obtained absorbance with the above diagram. The approximate H_2O_2 concentration can be monitored by using hydrogen peroxide detection strips (Quantifix). If the strip indicates a H_2O_2 concentration larger than 200 mg/L, the sample can be accordingly diluted. Always the quantity of the diluted sample should be 1 ml and it should be mixed with 1 ml of the standard PTO solution.

© 2019 by the authors. Submitted for possible open access publication under the terms and conditions of the Creative Commons Attribution (CC BY) license (http://creativecommons.org/licenses/by/4.0/).
